# Bibliographical Notices

**Published:** 1838-06-20

**Authors:** 


### BIBLIOGRAPHICAL NOTICES.

*The American Journal of Pharmacy, published by authority of the Philadelphia College of Pharmacy. Edited by* Joseph Carson, M. D., Professor of Materia Medica in the College, and Robert Bridges, M. D. Assisted by a Publishing Committee, &c. &c. &c. Vol. X. New Series, Vol. IV., No. 1, April, 1838. Philadelphia. 8 vo. pp. 88.

This is a truly valuable publication, and should be in the hands of every druggist in the country. Its tendency is of the most salutary description, enabling, at a trifling cost, and with little labour, the pharmacien to keep pace with the rapid improvements in his science, and avail himself of them.

This little Journal contains a vast amount of interesting and instructive matter, both original and selected. The communications are, in general, of no inconsiderable value, and exhibit a healthful and flattering picture of the condition of pharmacy amongst us. The analecta evidence judgment and practice in the selection.

The Journal of Pharmacy has, for a year past, been conducted by Professor Carson, assisted by several eminent collaborators, in a manner calculated to create a most favorable impression of his abilities and acquirements. We perceive, by an announcement in the last number, that Dr. Robert Bridges has been associated with Dr. Carson, in the editorship. Dr. Bridges brings to the task requisites of a high order; and is well known in this city for his ardent and successful devotion to chemical pursuits. He has our best wishes in his new vocation.

---

Nature and Treatment of Diseases of the Ear. By Dr. William Kramer. *Second Edition of the Author's Treatise on Chronic Deafness, much improved and enlarged. Translated from the German with the latest improvements of the author since the last London edition,* by James Risdon Bennett, M. D., Member of the Royal College of Physicians of London, &c. &c. &c. Philadelphia: Thomas, Cowperthwait & Co. 1838. 8vo. pp. 250.

Whilst Opthalmology has been prosecuted with unbounded zeal and assiduity, a singular and lamentable apathy has characterized the cultivation of acoustic medicine. A deplorable ignorance of its principles is avowed even by men whose reputation and attainments, in the other departments of their science, are deservedly great. The natural offspring of this conscious imbecility is an universal unwillingness to undertake the management of diseases of the ear, and, consequently, they are gladly abandoned to the mercy of the itinerant charlatan, who, from the credulity of the public, and the culpable sluggishness of the physician, pursues, unchecked, the even tenor of his way. The time, we trust, is not far distant, when the profession will be roused from this ignominious torpor, and apply themselves with earnestness and diligence to the study of aural maladies; when it will be deemed an integral part of the education of every enlightened practitioner; will attract a due consideration from every surgical teacher; and will constitute an important and essential qualification in the examination of every student.

The appearance of Dr. Kramer's Treatise has already contributed much to remove the opprobrium which has been so long attached to acoustic medicine, and its republication in this country will, we feel assured, contribute not a little to dissipate the profound ignorance in which it is still involved amongst us, and give a strong and salutary influence to its cultivation.

Fruitful as the present century has been of works in all the branches of our art, an unaccountable aridity and paucity distinguish that of aural medicine. Nor at any time has the subject received that attention its importance would seem imperatively to claim. Hippocrates mentions diseases of the ear only in connection with other complaints. Celsus was the first who assigned them an independent station, and offered some good rules for the management of acute inflammations of the ear, and counselled ocular inspection in chronic cases. Galen, although recognising etiological distinctions, recommends a practice founded on the crudest and most indiscriminating empiricism, and which was universally received for more than a thousand years. At the close of the fifteenth and commencement of the sixteenth century, Ingrassius, Eustachius, Fallopius, Vesalius, &c., elucidated the intricate subject of aural anatomy by a series of brilliant discoveries; but without any increase of pathological or therapeutic knowledge. In 1646, Fabricius Hildanus invented the speculum auris; but limited his investigations to the external meatus, and the adjacent parts. Bonet, Du Verney, Valsalva, Vieussens, and others, are more to be admired for the accuracy and minuteness of their anatomical descriptions, than for the luminousness or correctness of their pathological views, which are at best but theoretical subtleties. Two hundred years had now elapsed since the discovery of the Eustachian tube, and no endeavour had as yet been made to address any remedial agent to the ear through it, until 1724, when Guyot, a postmaster at Versailles, attempted it by the introduction of an instrument into it through the mouth. In 1741, Clelland, an Englishman, improved upon Guyot's procedure, by the introduction of a flexible silver catheter through the nose, which was subsequently perfected by the Montpelier physicians, by the substitution of an inflexible tube. At the end of the eighteenth century, perforation of the membrana tympani, as a cure for deafness, was lauded with great extravagance. Wright, Stevenson, Saunders, Buchanan, and Curtis are among the chief English writers on acoustic medicine. Not one of their productions unfortunately, are entitled to any credit. There is, in all of them, a deplorable absence of a genuine scientific and philosophical spirit; and, in general, exhibit a profound ignorance of the subject they treat upon. In France, the works of Saissy, Deleau, and Itard, have obtained considerable reputation. The former of these is nearly worthless; the two latter, especially that of Itard, merit more confidence, and possess intrinsic merit.

Dr. Kramer commences his *Treatise* with a critical review of otiatric literature, in which the reader will find much that is instructive and curious. We regret that we cannot offer any abstract of his critical remarks, obliged as we are to confine ourselves to a brief epitome of his views on the more practical parts of acoustic medicine.

Previous to entering into any investigation of the particular diseases of the ear, our author devotes a chapter to the consideration of the general as well as individual remedies which have been recommended, or attained celebrity, in their treatment. Before accompanying him in this survey, we shall state his opinion of one of those agents which he regards as exercising an important injurious influence on the auditory apparatus, differing as it does from the popular prejudice, though sanctioned by reason as well as fact.

"The application of *cold* in every form acts injuriously on the ear; not only on the auditory nerve, but even on the membranous constituent parts of the organ, whose small supply of blood, and of vital heat, is quite unequal to resist the power of cold. It is, therefore, a most pernicious prejudice, to think of invigorating the ear by washing it with cold water; it should be most carefully avoided. The ear should only be cleansed with tepid water; it should be guarded when bathing in fresh or salt water, or during cold affusions, not merely with cotton wool, but also by an oiled silk cap, and plunging overhead should be abstained from. Damp, cold, stormy weather is equally injurious, and from this the ear should be protected in precisely the same way." p. 29.

Dr. Kramer considers the prognosis in diseases of the ear as by no means bad. The peculiar structure of the organ, as well as its isolation, from the economy, forbid, it is true, very generally, the hope of any successful interposition of nature, "still it is of the utmost importance to know that diseases of the ear admit of a *very certain diagnosis*, (not, indeed, according to the old established mode,) that in general they run a very chronic course, and that, under the influence of these two circumstances, *they are almost all curable*, if the treatment *of them* be only undertaken in proper time and with the *proper remedies.*" The right moment is too often neglected, "and thus the disease is artificially, and quite contrary to its nature, rendered incurable."

Of the local remedies, which have obtained celebrity in the treatment of deafness, *Electricity*, *Galvanism*, and *Mineral Magnetism*, may be ranged together. Dr. Kramer does not think the efficacy of either of these agents, as yet established. No well authenticated case of permanent improvement, much less relief, having been promulgated. Neither does he attach any beneficial influence to the *moxa*, or *actual cautery*, so much vaunted, especially by Itard, in nervous deafness. *Blisters*, in slight cases, he does not consider as injurious; in obstinate diseases of the middle ear they are useless; and in nervous deafness, positively hurtful. In topical circumscribed inflammatory affections of the membrana tympani and of the meatus, where benefit might be anticipated from revulsion, he prefers the tartar emetic ointment, rubbed in below the mastoid process to avoid all risk of caries. This accident might, we think, be more effectually guarded against by the substitution of a milder irritant, whose action was less capricious; as the croton oil. *Issues* and *setons* he pronounces either useless, or positively mischievous. *Douches* within or without the ear, with a view of exciting the auditory nerve, whether of water or vapour, are absolutely dangerous. Particularly pernicious are *drops and injections*, which usually contain acid, and irritating ingredients. They constitute the popular empirical medicines in deafness. *Warm fomentations*, *injections of warm milk*, &c. &c., are perfectly innocuous, their application wasting time, when more energetic and appropriate remedies might be resorted to. *Topical bleeding* is alone demanded in acute inflammatory affections of the ear, and in such cases must be freely employed.

When we reflect on the limited and unimportant connection of the organ of hearing with the vascular and nervous systems, we cannot *a priori* anticipate any extrordinary benefit from the influence of general agents. Experience corroborates the hypothesis. *Russian vapour* baths have been highly commended, especially when the local affection was supposed to have originated in cold. Dr. Kramer has never witnessed the remotest actual relief result from their use. *Salt water* baths have frequently been prescribed, particularly in nervous deafness; positive injury is a most common consequence. The same may be said of warm sulphur, steel, and other baths, especially when any augmentation of vascular action exists. *Emetics* are nugatory in nervous deafness, and can only exercise a beneficial influence in obstructions of the Eustachian tube, when the mouth of it alone is closed. As auxiliary means in acute and chronic otitis, *purgatives* may be employed; in nervous deafness, by weakening the patient, they are baneful. *Bleeding* is admissible only on general principles. *Salivation*, *starvation*, and *inunction* "are never indicated in *any disease of the ear as such.*" Still, our author considers that careful attention should be paid to the general condition of the patient, not that this alone will effect the cure, but as preparing the way for the application of special treatment, and increasing the chances of ultimate success.

Dr. Kramer's classification, which is natural and lucid, is so arranged, that "particular diseases shall follow each other in the same order as that in which the constituent parts of the ear that are morbidly affected, are organically connected." His first division is into diseases of the middle, external, and internal ear.

The lesions of the external ear are much more common in childhood, during the development of the frame, than at any other period. They are: 1. Diseases of the auricle; *a*, erysipelatous inflammation; *b*, schirrous degeneration; *c*, furuncle. 2. Diseases of the Meatus externus; *a*, erysipelatous inflammation; *b*, inflammation of the glandular structure; *c*, inflammation of the cellular tissue; *d*, inflammation of the periosteum. 3. Diseases of the membrana tympani; *a*, acute inflammation, and perforation; *b*, chronic inflammation.

The influence of the auricle in the function of hearing has been variously estimated. Itard denies its having any use, whilst Buchanan attaches the highest importance to its integrity. The truth lies between these extremes, the auricle being neither indispensable, nor altogether immaterial to good hearing. As the affections of this part of the auditory apparatus, are neither distinctive, nor demand a different course of treatment than uniform lesions in similarly constituted organs, we shall not here enlarge upon them, but pass to the diseases of the meatus externus.

Our author dwells earnestly on the importance of ocular inspection in revealing the morbid changes which occur in the auditory passage, and the mebrana tympani. The speculum he deems indispensable to the aurist, and gives us a description, accompanied by a cut, of the one invented and used by himself. It resembles that of Itard, with the exception of the opening at the farther extremity being cylindrical, with a wider funnel-shaped orifice. The inner surface is coloured black, a polished surface confusing the examination, by reflecting the incident luminous rays.

Buchanan determines the soundness of the auditory organ by the perfection of the cerumenous secretion. This is opposed to all experience, there being often no deviation in the quantity or quality of the ear-wax in very many important diseases of the middle and internal ear. Diseases of the auditory canal is more common in childhood and youth, whilst manhood and old age are particularly liable to disorder of the middle and internal ear.

"Careful observation has taught me that all diseases of the auditory passage depend on inflammation of its organic constituent parts, which have a characteristic imprint, according as one or other of these organic parts is attacked. Diseases consequent upon these forms of inflammation, have no claim to be considered as independent diseases, but are naturally classed according to their origin." p. 86.

*Erysipelatous inflammation of the meatus externus*, is the common cause of those large and sudden accumulations of cerumen in the auditory passage, and which is often unjustly attributed to neglect and want of cleanliness on the part of the patient, but which, in reality, is a morbid product, whose removal no patient can affect, as the sensation of the canal, naturally acute, is heightened by the inflammatory action, and even at its anterior part will not endure the slightest touch. The prognosis is highly favourable, and the treatment simple and certain. The small syringes generally used, Dr. K. considers as inoperative, from their holding an insufficient supply of fluid, thus unnecessarily prolonging the operation; and the length and tenuity of the tube permitting it to be pushed too deep into the meatus of restless patients, occasioning unnecessary pain, and injuring the membrana tympani. He therefore employs an "ear-syringe, three inches in length, holding an ounce and a half of water, furnished with a pipe three quarters of an inch long, with an opening sufficiently large to allow of a powerful stream of water."* Simple lukewarm water alone need be employed, and will fulfil every indication. After the removal of the viscid cerumen has been accomplished, should any redness of the meatus be discovered, a solution, containing a grain of the acetate of lead to an ounce of water, may be dropped in, and in obstinate cases, counter irritation may be effected, by employing the tartar emetic ointment (or croton oil) behind the ear. Should there be evidence of ulceration of the meatus, it should be smeared with the tincture of myrrh or opium.

*Inflammation of the glandular structure of the meatus*, commonly known as *external catarrhal inflammation*, is the cause of the fleshy excrescences of the auditory canal. The disease, in its worst and most chronic form, never extends beyond the limits of the glandular structure. The most acrid secretion, though producing excoriation, never causes true ulceration. There is considerable redness and tumefaction of the auditory passage, with diminished calibre, extending to the entrance. After the first few days, there is a serous or muco-purulent discharge from the meatus, sometimes unirritating, yet often corrosive, varying in quantity, of a green or yellow tint, streaked with blood, dirty or whitish, and of a sweet or very offensive ammoniacal odour. Tinnitus occurs in most cases. After some time, the walls of the meatus are found covered by soft, spongy, vascular, vesicular, sensitive granulations, pedunculated or globular. These, though sometimes of a cartilaginous or bony hardness, spring from a broad base, are insensible, and of a pale red colour. The most frequent *exciting cause* of the disease, is the application of cold. Collections of indurated cerumen can never be classed as a cause. The prognosis is altogether favorable, where the inflammatory tumefaction is general, the disease yielding readily to a judicious and timely treatment. But where the glandular structure at the bottom of the meatus is alone involved—if the secretion be scanty or serous, the swelling insensate, and the polypi pale red, with a broad base, and hard, it may be viewed as a totally *incurable state*. The radical cure of pedunculated growths, as above described, is often tedious, the root remaining from which fresh vegetations spring. The only certain sign that the glandular integument is free from inflammatory excitement, is the re-appearance of the cerumenous secretion. In the treatment of the disease, cleanliness is of paramount importance, and the meatus must be syringed out several times a day, with lukewarm water. Sometimes cool, or cold water is more grateful to the patient; when such is the case, his wishes should be gratified. When the disease, as it often does, depends on constitutional causes, or cutaneous affections, the general principles of special therapeutics must be energetically employed.

* Ear syringes, of this construction, can be had of J. C. Turnpenny, druggist, N. E. corner of Spruce and Tenth streets.

When insects have entered the auditory passage, and elude the grasp of the forceps, sweet olive oil should be dropped into the ear, with the view of killing them; but no acrid, or irritating application should be used.

*Phlegmonous Inflammation* of the meatus, is of rare occurrence, and may be distinguished from the species just mentioned, by its invariably passing, as elsewhere, into suppuration. The absence of any carious, bony substance at the bottom of the abscess, prevents it being confounded with periostitis of the osseous portion of the auditory canal. The treatment should be such as will facilitate the formation of pus.

*Inflammation of the periosteum of the meatus externus*, is invariably followed by caries, and when the dead bone has been removed by exfoliation, the curative efforts of nature are often so powerful as to completely obliterate the canal for some lines in length. The prognosis is of a very gloomy character; the tendency of the strictured parts, after division, to reunite being considerable. Our author is unable to detail any complete cases, as none of his patients had sufficient perseverance to await the termination of the treatment.

Views, the most erroneous and gratuitous, have been assumed respecting the diseases of the membrana tympani. No hypothesis is more untenable than that of the relaxation of this membrane, as one of its morbid conditions. That of a preternatural tension is equally groundless. All its diseases are the result of inflammatory action. That rupture of the membrana tympani can occur as a primitive lesion, unpreceded by inflammation, our author denies, though vouched for by Du Verney, Leschevin, Itard, Saissy, and Curtis. Many authors still maintain the opinion that perforation of the membrana tympani does not necessarily impair the integrity of the auditory function; but this, Dr. Kramer thinks, arises from an imperfect method of comparison, there being no acknowledged invariable standard of admeasurement. Careful and frequent examinations have convinced him, though not entailing necessarily complete deafness, it is always followed by more or less dulness of hearing.

The only recognised diseases of the membrana tympani are, according to Dr. Kramer, those of an inflammatory nature, and their saquelæ, as opacity, thickening, perforation, purulent secretion, and polypous growths. The inflammatory nature of common *earach*, he thinks, is too often overlooked, and the patient, in consequence, subjected to a local irritative plan of treatment positively noxious. The curative means he employs to combat the affection, and which he represents as successful, are strictly antiphlogistic. In this, as well as the inflammatory disorders of the meatus, he appears to place great confidence in a solution of the acetate of lead—from one to ten grains to the ounce of water—dropped, three or four times a day, into the ear. The polypous growths which arise on the tympanal membrane, and usually have a short peduncle, he removes with a delicate double-edged knife, having a bent, sickle-shaped blade, and a blunt, rounded extremity, the remains of the parasite being touched with lunar caustic. "But the caustic is often far surpassed in efficacy by the acetate of lead, applied in the form of solution, as the reproductive power of the root of the polypus is thus, with most certainty, kept down." p. 131.

"If the membrana tympani be considerably thickened, quite insensible on being touched with a probe, and of cartilaginous hardness; and if, in consequence of this, the hearing has seriously suffered, there remains nothing for the improvement of the latter, but *perforation of the membrane.* Even in those cases, however, in which the operation is fairly indicated, it ought not to be had recourse to, excepting when both ears are affected in the same way, and suffer simultaneously from a high degree of difficulty of hearing; or, when the second ear, the tympanic membrane of which is not diseased, yet suffers from difficulty of hearing so incurable that perforation of the membrana tympani affords the only prospect of probable improvement. To this permission for the performance of the operation, I must, however, annex the following clause: that the most careful investigation of the ear to be operated on, must have proved that it is suffering from no other morbid condition, by which the success of the operation would be rendered fruitless." p. 132.

The veritable indication for the puncture of this membrane, is when it is preternaturally thickened, with entire absence of any co-existent aural affection.

The success of the operation depends on the permanence of the opening. Dr. Kramer prefers, to all other instruments, Himley's punch, which, by a gentle rotatory motion, removes a small circular portion of the membrane, and thus prevents subsequent occlusion. For the performance of the operation, the patient should be put in a strong light, with his head inclined to the opposite shoulder, to permit the solar rays to fall directly on the membrana tympani. The speculum is introduced into the meatus, with the left hand, and "the punch steadily and carefully conducted to the anterior and inferior third of the membrane, and then passed through with a gentle rotatory motion." (p. 137.) Unless cartilaginous degeneration has taken place, it readily yields, and usually a drop of blood alone issues. Should a puriform mucus flow from the puncture, and be found on the punch, disease of the middle ear exists, and no benefit will succeed the operation. Catgut bougies, &c. are all unnecessary to keep open the aperture.

Dr. Kramer concludes his chapter on the diseases of the membrana tympani with a section on *chronic inflammation*, which he regards, from the usual accompanying sound state of the meatus, as an idiopathic, independent affection. Its development is latent and mild, and consequently dangerous. The prognosis is very unfavourable, even under the most fortunate circumstances, it proving utterly intractable.

